# Prevalência das Complicações Cardiovasculares nos Indivíduos com Anemia Falciforme e Outras Hemoglobinopatias: Uma Revisão Sistemática

**DOI:** 10.36660/abc.20220207

**Published:** 2022-11-11

**Authors:** Andressa Lopes, Marina Tejo Dantas, Ana Marice Teixeira Ladeia

**Affiliations:** 1 Escola Bahiana de Medicina e Saúde Pública Salvador BA Brasil Escola Bahiana de Medicina e Saúde Pública, Salvador, BA – Brasil

**Keywords:** Anemia Falciforme, Complicações Cardiovasculares na Gravidez, Hemoglobinopatias

## Abstract

**Fundamento:**

A anemia falciforme (AF) é uma doença hereditária cujas complicações cardiovasculares são a principal causa de morte, o mesmo sendo observado em outras hemoglobinopatias. A identificação precoce dessas alterações pode modificar favoravelmente o curso da doença.

**Objetivo:**

Comparar a prevalência de complicações cardiovasculares entre indivíduos com AF e indivíduos com outras hemoglobinopatias.

**Métodos:**

Seguindo recomendações do protocolo PRISMA, realizou-se revisão sistemática da literatura com buscas nas bases de dados PubMed/Medline, associadas à busca manual. Incluídos estudos que analisaram a prevalência das alterações cardiovasculares nas hemoglobinopatias (AF, traço falciforme, hemoglobinopatia SC, alfatalassemia e betatalassemia). A qualidade metodológica dos artigos foi realizada pela escala de Newcastle-Ottawa.

**Resultados:**

Foram selecionados para análise quatro estudos, resultando em um tamanho amostral de 582 participantes: 289 portadores de AF, 133 possuem hemoglobinopatia SC, 40 com betatalassemia, 100 indivíduos saudáveis e nenhum com alfatalassemia ou traço falcêmico. Dilatação das câmaras cardíacas, hipertrofia ventricular esquerda e direita, hipertensão pulmonar, disfunção diastólica, insuficiência mitral e insuficiência tricúspide são mais prevalentes na AF do que nas demais hemoglobinopatias consideradas. A sobrecarga miocárdica de ferro é mais frequente na talassemia maior do que na AF. A função sistólica foi similar entre as hemoglobinopatias.

**Conclusão:**

Verificou-se maior comprometimento cardiovascular nos indivíduos com AF do que naqueles com as demais hemoglobinopatias, reforçando a necessidade de acompanhamento cardiovascular regular e frequente nos pacientes falcêmicos.

## Introdução

A redução da morbimortalidade dos indivíduos com doença falciforme advinda do avanço nas terapias específicas tem se tornado evidente. À medida que a idade desses pacientes aumenta, os efeitos crônicos da anemia hemolítica e episódios vaso-oclusivos levam a lesões crônicas de órgãos-alvo, destacando-se as complicações cardiovasculares,^1^ que são a principal causa de morte.^2^ Apesar do avanço terapêutico, a mortalidade nos adultos continua alta mesmo nos países desenvolvidos, com média de idade abaixo dos 50 anos.^3^

Ainda que as melhorias nos protocolos de transfusão sanguínea e de uso de agentes quelantes de ferro aumentaram a sobrevida dos pacientes com talassemia, a principal causa de morbidade e mortalidade nesses pacientes é a doença cardíaca,^4^ responsável por 75% das mortes.^5^ No Brasil, 10 a 20% dos indivíduos com talassemia dependentes de transfusão apresentam sobrecarga de ferro severa, com incidência de cardiopatias de 5%.^6^

Assim, diversas são as complicações cardiovasculares implicadas no curso clínico da anemia falciforme, bem como no das demais hemoglobinopatias. Este estudo visou comparar a prevalência de complicações cardiovasculares entre indivíduos com anemia falciforme e com outras hemoglobinopatias.

## Métodos

### Desenho do estudo

Revisão sistemática de literatura com busca norteada pela diretriz PRISMA, registrada no PROSPERO, sob o número CRD42021225542.

### Estratégia de busca

A busca de artigos foi realizada nas bases de dados PubMed/Medline, sendo aplicados os seguintes descritores consultados pelos sites *Medical Subject Headings* (MeSH) e Descritores em Ciências da Saúde (DeCS): “Sickle cell disease”, “Sickle Cell Anemia”, “ *Hemoglobinopathies”, “Hemoglobin SC Disease”, “Haemoglobin SC”, “Sickle Cell Trait”, “Beta-thalassemia”, “Alpha-thalassemia”, “Cardiac”, “Cardiovascular”.* Foi também realizada a busca manual dos artigos. Utilizou-se o operador boleano “AND” para agregar os descritores.

### Critérios de elegibilidade

Foram incluídos estudos observacionais e ensaios clínicos randomizados e não randomizados, que atendessem o critério de analisar a prevalência das alterações cardiovasculares nas seguintes hemoglobinopatias (anemia falciforme, traço falciforme, hemoglobinopatia SC, alfatalassemia e betatalassemia). Incluídos artigos em inglês e português, publicados entre fevereiro de 2011 e fevereiro de 2021. Foram excluídas: publicações duplicadas, revisões sistemáticas e metanálises, relatos de caso, relatos de série e estudos em animais.

### Identificação e seleção de estudos

Dois autores analisaram, separadamente, o título e o resumo de cada trabalho, identificando quais preenchiam os critérios de inclusão. Um terceiro pesquisador avaliou os artigos em que houve discordância, completando a seleção de artigos elegíveis para leitura integral. Posteriormente, foi feita a leitura completa de cada estudo por um dos autores, a fim de assegurar os critérios da revisão sistemática, até se chegar à lista final dos trabalhos incluídos na revisão.

### Extração e análise de dados

Os dados extraídos foram: título, autor, ano de publicação, desenho, período e local de realização do estudo, tamanho amostral e objetivos. As variáveis hipertensão pulmonar, disfunção diastólica e sistólica do ventrículo esquerdo, disfunção ventricular direita, presença de insuficiência mitral e de insuficiência tricúspide foram analisadas.

### Qualidade metodológica

A qualidade metodológica dos estudos foi avaliada pela escala de Newcastle-Ottawa, ferramenta indicada para análise de estudos de coorte e de caso-controle. A pontuação da qualidade metodológica dos estudos de coorte foi calculada em três componentes: seleção dos grupos (0 – 4 pontos), qualidade de ajuste para confusão (0 – 2 pontos) e avaliação do desfecho (0 – 3 pontos). Nos estudos de caso-controle, foi avaliada a seleção dos grupos (0 – 4 pontos), a qualidade de ajuste para confusão (0 – 2 pontos) e a exposição (0 – 4 pontos). A pontuação máxima é de 9 pontos, representando alta qualidade metodológica. Dois pesquisadores independentes julgaram a qualidade/risco de viés dos trabalhos.

## Resultados

### Identificação e seleção dos estudos

**Figura 1:**
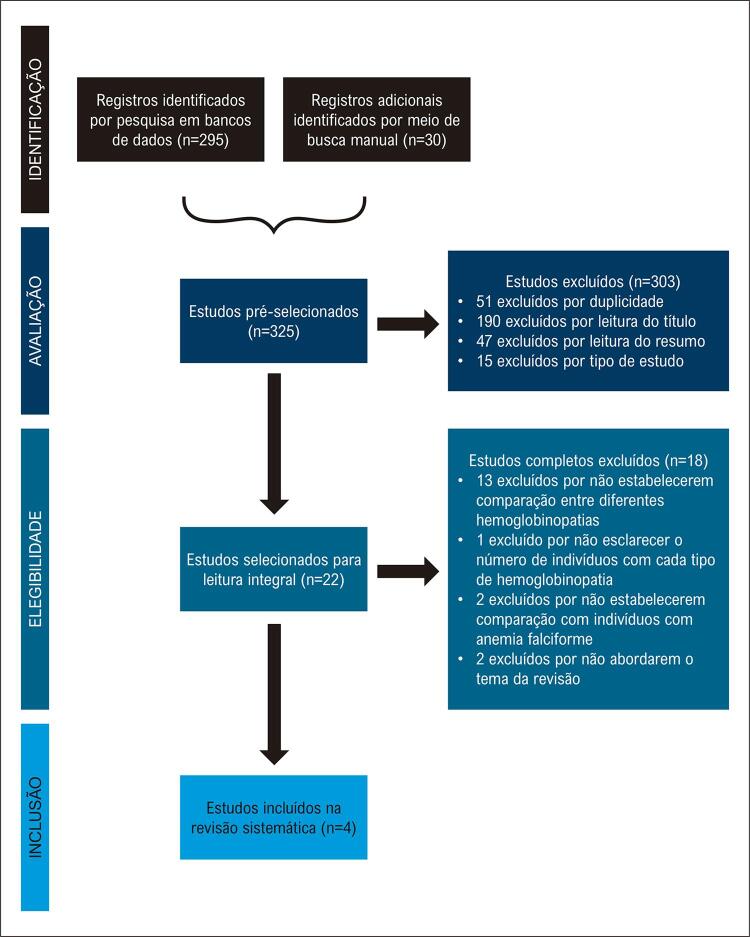
Fluxograma de seleção dos estudos.

A partir do banco de dados eletrônicos e da busca manual, 325 artigos foram identificados. Após remoção de artigos duplicados e seleção pela leitura de títulos, resumos e textos completos, obteve-se 4 artigos incluídos na síntese qualitativa do trabalho. A seleção dos estudos está representada no fluxograma na Figura 1 .

### Características gerais dos estudos

Dos quatro artigos selecionados, três são estudos de coorte e um é caso-controle. Os anos de publicação variaram de 2016 a 2019. O tamanho amostral variou de 110 a 180 participantes, totalizando 582 participantes: 289 portadores de anemia falciforme, 133 com hemoglobinopatia SC, 40 com betatalassemia, 100 indivíduos saudáveis e nenhum com alfatalassemia ou traço falcêmico. Vinte indivíduos possuíam outros genótipos de doença falciforme que não preenchem os critérios de inclusão deste estudo. A Tabela 1 apresenta as características gerais dos estudos.

**Tabela 1:** Características gerais dos estudos selecionados

Autor	Ano de publicação	Título	Período de realização	Local de realização	Desenho do estudo	Tamanho amostral	Objetivos
Philippe M. Adjagba, Gaston Habib, Nancy Robitaille, et al.	2016	Impact of sickle cell anemia on cardiac chambre size in the paediatric population	Não mencionado	Canadá	Coorte restrospectiva	n = 110	Descrever a extensão das anormalidade miocárdicas e determinar os índices hematológicos que poderiam afetar principalmente a função cardíaca nos pacientes com doença falciforme
Jamie K. Harrington, Usha Krishnan, Zhezhen Jun, et al.	2017	Longitudinal analysis of echocardiographic abnormalities in children with sickle cell disease	1994-2013	Estados Unidos	Coorte retrospectiva	n =172	Identificar parâmetros clínicos e laboratoriais associados ao desenvolvimento de anormalidade cardíacas
Paul Guedeney, Fraçois Lionnet, Alexandre Ceccaldi, et al	2018	Cardiac manifestations in sickle cell disease varies with patient genotype	Maio 2008 Maio 2015	França	Coorte retrospectiva	n = 180	Descrever o remodelamento cardíaco e suas correlações em pacientescom HbSC e comparar com os pacientes com anemia falciforme e com os indivíduos saudáveis
Antonie Fakhry Abdel Massih, Khaled M. Salama, Carolyne Ghobrial, et al	2019	Discrepancy in patterns of myocardial involvement in beta-thalassaemia vs. Sickle cell anemia	Abril 2017 Out 2018	Egito	Caso controle	n = 120	Comparar a mecânica ventricular esquerda nos pacientes com talassemia e nos com anemia falciforme através da “ecocardiografia com strain”

## Resultados

Adjagba et al. constataram que, embora a dilatação ventricular direita tenha sido similar entre os pacientes HbSS e os HbSC, a dilatação ventricular esquerda foi mais frequente na AF do que na hemoglobinopatia SC, tendo sido observada em 51,4% vs 24,2% dos pacientes, respectivamente [OR=2,1(1,11−4,03)] , o mesmo ocorrendo com a dilatação de ambos ventrículos, presente em 38,9% x 12,5% dos pacientes com cada genótipo, respectivamente [OR=3,4(1,19−8,13)] Não se observou diferenças significativas entre os genótipos na frequência de disfunção miocárdica esquerda medida pela fração de encurtamento do ventrículo esquerdo e pela relação E/e`. Verificou-se hipertrofia ventricular esquerda (HVE) em 25% dos pacientes com AF, dado não constatado na amostra com HbSC.

Harrington et al. avaliaram 829 ecocardiogramas realizados em 172 pacientes, sendo observada uma incidência cumulativa das anormalidades ecocardiográficas. A idade média do primeiro eletrocardiograma foi 8,74 ± 3,49 anos de idade (variando entre 5,12 a 19,7 anos de idade), com uma média de 4,82 ± 3,06 estudos realizados por paciente por um período de 6.88 ± 5.16 anos. A distribuição por idade do primeiro ecocardiograma foi: 78 (45,4%) na idade de 5 a 7 anos ou menos, 72 (41,8%) na idade acima de 7 a 13 anos ou menos e 22 (12,8%) acima de 13 anos de idade. HVE, diâmetro sistólico final do ventrículo esquerdo (DSFVE) e diâmetro diastólico final do ventrículo esquerdo (DDFVE) aumentados foram encontradas em uma idade mais precoce do que a velocidade de regurgitação tricúspide (VRT) anormal, esta última encontrada principalmente na infância tardia e na adolescência. A prevalência das anormalidades ecocardiográficas foi 25%, 41%, 58%, 7% e 25% para HVE, aumento DSFVE e DDFVE, redução da FE de VE e aumento de VRT, respectivamente. Além disso, os pacientes com HbSS e HbSβ^0^-talassemia tiveram 8,04% mais chances de apresentar HVE, 8,37% mais chances de apresentar dilatação de VE ao final da sístole e 11,9% mais chances de apresentar dilatação de VE ao final da diástole. A chance de desenvolver aumento da velocidade de regurgitação tricúspide e diminuição da fração de encurtamento de VE foram similares entre os genótipos envolvidos no estudo.

Guedeney et al. compararam o remodelamento cardíaco entre indivíduos com as hemoglobinopatias HbSS e HbSC e indivíduos saudáveis, envolvendo 180 pacientes. A dilatação de VE foi maior nos pacientes com HbSS do que nos indivíduos com HbSC [ $\mathrm{DDFVE}/\mathrm{SC}\;=\;32\;\mathrm{mm}/m^{2}\;(\mathrm{IIQ}:\;29-33)\;x\;28\;\mathrm{mm}/m^{2\;}(\mathrm{IIQ}:\;26-30)$ , respectivamente, p < 0,0001; $\mathrm{VDFVE}/\mathrm{SC}\;=\;91\;\mathrm{mL}/m^{2}\;(\mathrm{IIQ}:\;73-105)\;x\;64\;\mathrm{mL}/m^{2}\;(\mathrm{IIQ}:\;54-72)$ , respectivamente, p < 0,001], o mesmo ocorrendo com AE [ $\mathrm{VAE}/\mathrm{SC}\;=\;49\;\mathrm{mL}/m^{2}\;(\mathrm{IIQ}:\;42-60)\;x\;33\;\mathrm{mL}/m^{2}\;(\mathrm{IIQ}:\;30-38)$ respectivamente, p < 0,001]. Da mesma forma, HVE foi mais frequente na AF do que na HbSC [ $\mathrm{MVE}/\mathrm{SC}\;=\;101\;g/m^{2}\;(\mathrm{IIQ}:\;84-115)\;x\;76\;g/m^{2}\;(\mathrm{IIQ}:\;65-87)$ , p < 0,001; MVE/A = 39 g/m (IIQ: 24-48) x 32 g/m (IIQ: 28-36) , p < 0,001], independentemente do método de indexação (superfície corpórea ou altura), ressaltando-se que a HVE foi principalmente excêntrica. Nos pacientes com HbSS, observou-se aumento da pressão arterial sistólica pulmonar – avaliada pela VRT – em 32 (53%) pacientes, sendo similar entre pacientes HbSC e controles. A disfunção diastólica de VE foi mais prevalente na AF do que nos indivíduos com HbSC e nos saudáveis (p = 0,04). A fração de ejeção do ventrículo esquerdo (FEVE) foi similar nos três grupos.

AbdelMassih et al. avaliaram o padrão de envolvimento miocárdico em 120 pacientes em estudo de caso-controle. O T2* miocárdico foi mais indicativo de sobrecarga miocárdica de ferro nos pacientes com betatalassemia maior do que naqueles com AF (T2* miocárdico = 16,6 ± 1,8 ms; 25,5 ± 2,2 ms, respectivamente). O *strain global longitudinal* (SGL) foi semelhante entre os pacientes com betatalassemia maior e os com AF, porém ambos grupos apresentaram menores valores de SGL quando comparados com os indivíduos saudáveis (SGL = -15 ± 1,6%; -21,5 ± 1,9%,indivíduos com betatalassemia maior e saudáveis, respectivamente; SGL = -15 ± 1,2%; -21,5 ± 1,9%, indivíduos com AF e saudáveis, respectivamente). Houve diferença entre os grupos de hemoglobinopatias quando se avaliou o SGL epicárdico e endocárdico: o SGL epicárdico foi mais baixo nos pacientes com betatalassemia maior (SGL epicárdico = -10,9 ± 2%; -19,9 ± 1,7%, na betatalassemia maior e na AF, respectivamente), o SGL endocárdico foi mais baixo nos pacientes falcêmicos (SGL endocárdico = -19,95 ± 1,7%; -10,65 ± 1,6%, na betatalassemia maior e na AF, respectivamente). Verificou-se que a função sistólica pela FEVE avaliada pelo modo M e pela fração de encurtamento de VE foi normal e similar nos 3 grupos de pacientes (FEVE = 73,2 ± 3,3 %; 71,2 ± 1,7;72,4 ± 2,9, no grupo de betatalassemia maior, AF e indivíduos saudáveis, respectivamente; fração de encurtamento de VE = 35,5 ± 2%; 35,5 ± 0,98%; 37,5 ± 3,3%, no grupo de betatalassemia maior, AF e indivíduos saudáveis, respectivamente), o mesmo sendo observado para a função diastólica de VE pela relação E/e´ (E/e´ = 6,89 ± 2; 6,6 ± 1,9; 6,52 ± 1,49, no grupo de betatalassemia maior, AF e indivíduos saudáveis, respectivamente). A FEVE avaliada pelo modo 3D foi menor nos pacientes com AF e do que nos controles ( FEVE = 62% ± 11,2 x 66% ± 13,2 , respectivamente) e também menor nos pacientes com betatalassemia maior do que nos controles ( FEVE = 61% ± 10,1 x 66% ± 13,2 , respectivamente), sendo similar nas duas hemoglobinopatias. Os principais resultados se encontram na Tabela 2 .

**Tabela 2:** Principais resultados dos estudos selecionados

Autor/ano de publicação	População	Variável cardiovascular analisada	Alteração cardiovascular encontrada	Conclusão
Philippe M. Adjagba et al. (2016)	110 pacientes com Doença Falciforme (72 HbSS; 32 HbSC; 6 HbSβ – talassemia)	VVD VVE MVE FE de VE Relação E/e´ IPM	DVD DVE Disfunção diastólica Disfunção sistólica Anormalidade na MVE	DVE foi maior nos pacientes com anemia falciforme (HbSS) do que nos pacientes com hemoglobinopatia SC (HbSC); HVE foi observada apenas nos pacientes com anemia falciforme (HbSS) e a anormalidade na MVE foi mais prevalente nesse grupo de pacientes; DVD, FE de VE e relação E/e´ foram similares entre os pacientes com anemia falciforme (HbSS) e os indivíduos com hemoglobinopatia SC (HbSC).
Jamie K. Harrington et al. (2017)	172 pacientes com Doença Falciforme (117 HbSS; 41 HbSC; 5 HbSβᴼ – talassemia; 9 HbSβ⁺ - talassemia)	MVE DSFVE DDFVE FE de VE VRT	HVE DVE ao final da sístole e ao final da diástole ↓FE de VE ↑VRT	Pacientes com genótipo HbSS e HbSβ0-talassemia foram mais propensos a desenvolver HVE, DVE ao final da sístole e ao final da diástole. A chance de desenvolver aumento da VRT e diminuição da FE de VE foram similares entre todos os genótipos envolvidos no estudo.
Paul Guedeney et al. (2018)	120 pacientes com Doença Falciforme (60 HbSS; 60 HbSC) e 60 pacientes saudáveis	DDFVE/SC MVE/SC MVE/A VDFVE/SC IC VRT FEVE Onda EM Onda A Relação E/A TD Onda e´ Relação E/e´ VAE/SC	DVE ao final da diástole DAE HVE ↑IC ↑VRT ↑Relação E/e´ Disfunção diastólica de VE	DAE, DVE e IC foram maiores nos pacientes HbSS do que nos pacientes HbSC e do que nos controles; HVE, aumento da VRT e disfunção diastólica de VE foram mais frequentes nos pacientes HbSS do que nos pacientes HbSC e nos controles (pacientes HbSS tiveram maiores: onda E, relação E/A, TD, onda e´, relação E/e´); DAE, DVE, MVE/SC, MVE/A, relação E/e´ foram maiores nos pacientes HbSC do que nos controles; Onda e´ foi menor nos pacientes HbSC do que nos controles; IC e VRT foram similares entre os pacientes HbSC e os controles; FEVE foi similar entre os 3 grupos.
Antoine Fakhry AbdelMassih et al. (2019)	40 pacientes com Anemia Falciforme (HbSS), 40 pacientes com Betatalassemia Maior (β^0^/β^0^) e 40 pacientes saudáveis	FEVE FE de VE Relação E/e´ SGL SGL epicárdico SGL endocárdico T2* miocárdico	Sobrecarga miocárdica de ferro ↓SGL Disfunção subendocárdica Disfunção subepicárdica	T2* miocárdico foi maior nos pacientes com betatalassemia maior do que nos pacientes com anemia falciforme; SGL foi semelhante entre os pacientes com betatalassemia maior e aqueles com anemia falciforme, porém ambos os grupos de pacientes tiveram SGL reduzido em comparação com os indivíduos saudáveis; SGL epicárdico foi mais baixo nos pacientes com betatalassemia maior do que nos pacientes com anemia falciforme; SGL endocárdico foi mais baixo nos pacientes com anemia falciforme do que nos pacientes com betatalassemia maior; A função sistólica e a função diastólica de VE foram normais e similares entre os 3 grupos.

DAE: dilatação do átrio esquerdo; DDFVE: diâmetro diastólico final do ventrículo esquerdo; DDFVE/SC: diâmetro diastólico final do ventrículo indexado por superfície corpórea; DSFVE: diâmetro sistólico final do ventrículo esquerdo; DVD: dilatação do ventrículo direito; DVE: dilatação do ventrículo esquerdo; FE: fração de encurtamento; FEVE: fração de ejeção do ventrículo esquerdo; HVE: hipertrofia ventricular esquerda; IC: índice cardíaco; IPM: índice de performance miocárdica; MVE: massa ventricular esquerda; MVE/A: massa ventricular esquerda indexada por altura; MVE/SC: massa ventricular esquerda indexada por superfície corpórea; Onda A: onda da contração atrial no fluxo mitral; Onda E: onda do enchimento rápido no fluxo mitral; Onda e´: onda da velocidade diastólica precoce por doppler tissular; Onda EM: onda do enchimento rápido no fluxo mitral do anel mitral; Relação E/A: relação entre as ondas E e A no fluxo mitral; Relação E/e´: relação entre a onda E no fluxo mitral e a onda e´ por doppler tissular; SGL: strain global longitudinal; SGLVD: strain global longitudinal do ventrículo direito; SGLVE: strain global longitudinal do ventrículo esquerdo; TD: tempo de desaceleração; T2*: relaxometria miocárdica da ressonância magnética cardíaca; VDFVE/SC: volume diastólico final do ventrículo esquerdo indexado por superfície corpórea; VE: ventrículo esquerdo; VRT: velocidade de regurgitação tricúspide; VVD: volume do ventrículo direito; VVE: volume do ventrículo esquerdo. Todos os artigos adotaram nível de significância estatística de 5%.

### Risco de viés dos estudos selecionados

A qualidade metodológica dos estudos incluídos nesta revisão foi alta. Dos estudos de coorte, um obteve oito pontos na escala de Newcastle-Ottawa e dois obtiverem nove pontos na mesma escala. O estudo de caso-controle obteve 8 pontos na escala empregada.

## Discussão

As complicações cardiovasculares são a principal responsável pela morbimortalidade nos pacientes com HbSS. Ressalta-se o papel do ecocardiograma para a identificação precoce das alterações cardíacas nesses pacientes, tal como foi evidenciado pelos achados do presente trabalho. Assim, constatou-se a maior prevalência de hipertrofia ventricular, dilatação das câmaras cardíacas, disfunção diastólica, insuficiência mitral e tricúspide e hipertensão pulmonar nos indivíduos com anemia falciforme em comparação com aqueles com as demais hemoglobinopatias consideradas neste estudo.

A dilatação das câmaras cardíacas, principalmente do VE decorre do remodelamento miocárdico compensatório em resposta à anemia crônica.^7 - 12^ A análise de associações entre variáveis ecocardiográficas em indivíduos falcêmicos demonstrou que indivíduos com maior DDFVE/SC apresentaram maiores valores de VAE/SC e de VRT, bem como menor FEVE, indicando disfunção sistólica esquerda com repercussão em câmaras direitas.^2 , 10^ A HVE foi independentemente associada a alterações dos parâmetros ecocardiográficos de disfunção diastólica, como diminuição do tempo de desaceleração da velocidade de influxo mitral precoce, aumento da relação E/e´ e aumento da velocidade de regurgitação tricúspide, que pode ser explicado pela redução da complacência ventricular esquerda nesses pacientes.

A disfunção diastólica está entre as principais alterações cardiovasculares relatadas na doença falciforme, sendo a frequência desse achado dependente dos parâmetros ecocardiográficos utilizados para avaliar a função diastólica, da idade do paciente e de comorbidades associadas.^9^ Vasconcelos et al.^9^ explicaram a ocorrência de função diastólica normal nos indivíduos com doença falciforme como resultado de uma idade jovem (média da idade de 26,5 anos), ausência de comorbidades e utilização do doppler tissular, cuja maior especificidade decorre de sua capacidade de medir as velocidades miocárdicas, não sofrendo alterações com mudanças da pré-carga.^13^

A associação verificada por Whipple et al.^14^ entre e´M e e´T diminuídos e SLGVE e SLGVD também diminuídos, sugere que a prevalência aumentada de disfunção diastólica nas crianças com doença falciforme reduz a deformabilidade miocárdica, medida pelo SLG. Em pacientes com HbSC, enquanto Adjagba et al.^7^ observaram uma relação E/e´ similar entre esses pacientes e os indivíduos com HBSS, Guedeney et al.^15^ constataram maior frequência de disfunção diastólica ventricular esquerda nos pacientes com AF e hipertensão arterial sistêmica, o que corrobora a hipótese sugerida por Desai et al.^8^ de que o comprometimento da função diastólica nesse grupo de pacientes decorre da pós-carga aumentada. Esses dados sugerem que a disfunção diastólica é frequente, precoce e de provável etiologia multifatorial em indivíduos com AF.

Nos pacientes com doença falciforme, a função sistólica encontra-se normalmente preservada. Contudo, já foi demonstrada significativa prevalência de função sistólica ventricular esquerda baixa em pacientes com HbSS e com HbSC.^7^ Marcador precoce da disfunção sistólica, o SGL mede a deformabilidade miocárdica e, o aumento dos seus valores indica a existência de uma condição de base alterando a deformabilidade miocárdica como mecanismo compensatório. Ao avaliar a associação do SGL com medidas tradicionais de função sistólica ventricular – FEVE e ESPAT – nas crianças com doença falciforme, Whipple et al.^14^ demonstraram concordância entre tais variáveis: SGLVE e SGLVD diminuídos associados com FEVE e ESPAT também diminuídos. A diminuição da ESPAT reflete a função sistólica prejudicada de VD. Como a função sistólica de VE está geralmente preservada na doença falciforme, a ESPAT anormal pode indicar elevação crônica das pressões pulmonares. Outrossim, o SGLVD mostrou-se prejudicado pela elevada pressão pulmonar e pela disfunção diastólica do VD.^12^

Na comparação entre AF e betatalassemia maior, o estudo de caso-controle incluído^16^ demonstrou predominância de disfunção subendocárdica na AF e de disfunção subepicárdica na betatalassemia maior, explicada pela alta vascularidade do epicárdio com consequente deposição de ferro. O T2* miocárdico foi fortemente correlacionado com o SGL epicárdico, mas não com o SGL endocárdico. Por sua vez, a diminuição do SGL subendocárdico verificado na AF justifica-se pela doença microvascular nesses pacientes, caracterizada por possível isquemia microvascular subendocárdica, por meio da depleção de NO e sugerida pelo aumento de LDH.

A respeito dos parâmetros para avaliar a função sistólica, vale ressaltar que, no mesmo estudo, as medidas da FEVE diferiram de acordo com o método utilizado: quando avaliada pelo modo-M, a FEVE foi similar entre os 3 grupos, porém, quando analisada pela ecocardiografia 3D, a FEVE mostrou ser menor nos indivíduos com AF do que nos saudáveis e similar na comparação com aqueles com betatalassemia maior.

A função ventricular direita é comumente avaliada por meio da VRT e da excursão sistólica do plano do anel tricúspide. A VRT esteve incluída entre os preditores de eventos adversos no trabalho de Vasconcelos et al.^9^ Outrossim, VRT ≥ 2,5 m/s foi preditor de mortalidade dentro de 3 anos por Damy et al.^10^ Neste último trabalho, VRT elevada foi associada com FEVE mais baixa e com VAE/SC mais alto, alterações comumente associadas a altas pressões de enchimento e ao risco de hipertensão pulmonar pós-capilar.

Ressalta-se que a maioria dos estudos foi realizada com amostras relativamente pequenas. Além disso, as variáveis cardiovasculares analisadas diferiram nos estudos incluídos. A despeito das limitações, a presente revisão deve ser considerada um instrumento de atualização sobre uma patologia de comprometimento sistêmico, permitindo a melhor compreensão das alterações cardiovasculares nos diferentes genótipos de hemoglobinopatias.

## Conclusão

A prevalência de complicações cardiovasculares como dilatação das câmaras cardíacas, HVE e HVD, hipertensão pulmonar, disfunção diastólica, insuficiência mitral e insuficiência tricúspide são maiores nos pacientes com AF do que nos indivíduos com as demais hemoglobinopatias consideradas neste estudo. Globalmente, não houve diferenças entre a função sistólica dos pacientes com AF e a daqueles com as demais hemoglobinopatias.

## References

[1] Gladwin MT (2016). Cardiovascular Complications and Risk of Death in Sickle-cell Disease. Lancet.

[2] Hammoudi N, Lionnet F, Redheuil A, Montalescot G (2020). Cardiovascular Manifestations of Sickle Cell Disease. Eur Heart J.

[3] Rai P, Niss O, Malik P (2017). A Reappraisal of the Mechanisms Underlying the Cardiac Complications of Sickle Cell Anemia. Pediatr Blood Cancer.

[4] Dimitroglou Y, Anagnostopoulos F, Aggeli C, Delicou S, Xydaki A, Patsourakos D (2020). Severity of Heart Failure and Health-related Quality of Life in Beta-thalassemia Patients: A Cross-sectional Study. Ann Hematol.

[5] Paul A, Thomson VS, Refat M, Al-Rawahi B, Taher A, Nadar SK (2019). Cardiac Involvement in Beta-thalassaemia: Current Treatment Strategies. Postgrad Med.

[6] Ministério da Saúde (2016). Orientações para o Diagnóstico e Tratamento das Talassemias Beta.

[7] Adjagba PM, Habib G, Robitaille N, Pastore Y, Raboisson MJ, Curnier D (2017). Impact of Sickle Cell Anaemia on Cardiac Chamber Size in the Paediatric Population. Cardiol Young.

[8] Desai AA, Patel AR, Ahmad H, Groth JV, Thiruvoipati T, Turner K (2014). Mechanistic Insights and Characterization of Sickle Cell Disease-associated Cardiomyopathy. Circ Cardiovasc Imaging.

[9] Vasconcelos MC, Nunes MC, Barbosa MM, Fernandes BM, Passaglia LG, Silva CM (2015). Left Ventricular Remodeling in Patients with Sickle Cell Disease: Determinants Factors and Impact on Outcome. Ann Hematol.

[10] Damy T, Bodez D, Habibi A, Guellich A, Rappeneau S, Inamo J (2016). Haematological Determinants of Cardiac Involvement in Adults with Sickle Cell Disease. Eur Heart J.

[11] Harrington JK, Krishnan U, Jin Z, Mardy C, Kobsa S, Lee MT (2017). Longitudinal Analysis of Echocardiographic Abnormalities in Children With Sickle Cell Disease. J Pediatr Hematol Oncol.

[12] Chiadika S, Lim-Fung M, Llanos-Chea F, Serauto Canache A, Yang W, Paruthi C (2018). Echocardiographic Parameters to Identify Sickle Cell Patients with Cardio-pathology. Echocardiography.

[13] Pedone M, Castro I, Feier F, Pandolfo F (2003). Doppler Tissular na Avaliação da Função Diastólica Ventricular Esquerda e Variações com a Idade.

[14] Whipple NS, Naik RJ, Kang G, Moen J, Govindaswamy SD, Fowler JA (2018). Ventricular Global Longitudinal Strain is Altered in Children with Sickle Cell Disease. Br J Haematol.

[15] Guedeney P, Lionnet F, Ceccaldi A, Stojanovic KS, Cohen A, Mattioni S (2018). Cardiac Manifestations in Sickle Cell Disease Varies with Patient Genotype. Br J Haematol.

[16] AbdelMassih AF, Salama KM, Ghobrial C, Haroun B, Rahman MA (2020). Discrepancy in Patterns of Myocardial Involvement in Beta-thalassaemia vs. Sickle Cell Anaemia. Acta Cardiol.

