# Supplementary material for: Prevalência das Complicações Cardiovasculares nos Indivíduos com Anemia Falciforme e Outras Hemoglobinopatias: Uma Revisão Sistemática
Source: Arq Bras Cardiol. 2022 Nov 11;119(6):893–9. [Article in Portuguese] doi: 10.36660/abc.20220207 (PMC9814804; doi:10.36660/abc.20220207)
Supplement: Translation

# Translation: Prevalence of Cardiovascular Complications in Individuals with Sickle Cell Anemia and Other Hemoglobinopathies: A Systematic Review

**Authors:** Andressa Lopes, Marina Tejo Dantas, Ana Marice Teixeira Ladeia

**Affiliations:** 1 Brazil Escola Bahiana de Medicina e Saúde Pública, Salvador, BA – Brazil

**Keywords:** Anemia, Sickle Cell, Pregnancy Complications, Cardiovascular, Hemoglobinopathies

## Abstract

**Background:**

Sickle cell anemia (SCA) is a hereditary disease whose cardiovascular complications are the main cause of death, the same being observed in other hemoglobinopathies. Early identification of these changes can favorably modify the course of the disease.

**Objective:**

To compare the prevalence of cardiovascular complications between individuals with SCA and individuals with other hemoglobinopathies.

**Method:**

Following the recommendations of the PRISMA protocol, a systematic literature review was carried out with searches in PubMed/Medline databases, associated with a manual search. Studies that analyzed the prevalence of cardiovascular alterations in hemoglobinopathies (SCA, sickle cell trait, SC hemoglobinopathy, alpha-thalassemia and beta-thalassemia) were included. The methodological quality of the articles was assessed using the Newcastle-Ottawa scale.

**Results:**

Four studies were selected for analysis, resulting in a sample size of 582 participants: 289 with SCA, 133 with SC hemoglobinopathy, 40 with beta-thalassemia, 100 healthy individuals and none with alpha-thalassemia or sickle cell trait. Dilatation of the cardiac chambers, left and right ventricular hypertrophy, pulmonary hypertension, diastolic dysfunction, mitral regurgitation and tricuspid regurgitation are more prevalent in SCA than in the other hemoglobinopathies considered. Myocardial iron overload is more frequent in thalassemia major than in sickle cell anemia. Systolic function is similar between different hemoglobinopathies.

**Conclusion:**

There is greater cardiovascular impairment in individuals with SCA than in those with other hemoglobinopathies, reinforcing the necessity for regular cardiovascular follow-up in sickle cell patients.

## Introduction

The reduction in morbidity and mortality of individuals with sickle cell disease resulting from advances in specific therapies has become evident. As the age of these patients increases, the chronic effects of hemolytic anemia and vaso-occlusive episodes lead to chronic lesions of target organs, especially cardiovascular complications,^1^ which are the main cause of death.^2^ Despite therapeutic advances, adult mortality remains high even in developed countries, with a mean age below 50 years.^3^

Although improvements in blood transfusion protocols and the use of iron chelating agents have increased the survival of patients with thalassemia, the main cause of morbidity and mortality in these patients is heart disease,^4^ responsible for 75% of deaths.^5^ In Brazil, 10 to 20% of transfusion-dependent individuals with thalassemia have severe iron overload, with an incidence of heart disease of 5%.^6^

Thus, there are several cardiovascular complications involved in the clinical course of sickle-cell anemia, as well as in that of other hemoglobinopathies. This study aimed to compare the prevalence of cardiovascular complications among individuals with sickle-cell anemia and other hemoglobinopathies.

## Methods

### Study design

Systematic literature review with a search guided by the PRISMA guideline, registered in PROSPERO, under the number CRD42021225542.

### Search strategy

The search for articles was performed in the PubMed/Medline databases, using the following descriptors consulted by the Medical Subject Headings (MeSH) and Health Sciences Descriptors (DeCS) websites: “Sickle cell disease”, “Sickle Cell Anemia”, “Hemoglobinopathies”, “Hemoglobin SC Disease”, “Haemoglobin SC”, “Sickle Cell Trait”, “Beta-thalassemia”, “Alpha-thalassemia”, “Cardiac”, “Cardiovascular”. A manual search of the articles was also performed. The Boolean operator “AND” was used to aggregate the descriptors.

### Eligibility criteria

Observational studies and randomized and non-randomized clinical trials that met the criteria of analyzing the prevalence of cardiovascular changes in the following hemoglobinopathies (sickle cell anemia, sickle cell trait, SC hemoglobinopathy, alpha-thalassemia and beta-thalassemia) were included. Articles in English and Portuguese published between February 2011 and February 2021 were included. Duplicate publications, systematic reviews and meta-analyses, case reports, series reports and animal studies were excluded.

### Identification and selection of studies

Two authors separately analyzed the title and the abstract of each work, identifying which ones met the inclusion criteria. A third researcher evaluated the articles in which there was disagreement, completing the selection of articles eligible for full reading. Subsequently, a complete reading of each study was performed by one of the authors, in order to ensure the criteria of the systematic review, until reaching the final list of works included in the review.

### Data extraction and analysis

The extracted data were: title, author, year of publication, design, period and place of study, sample size and objectives. The variables pulmonary hypertension, left ventricular diastolic and systolic dysfunction, right ventricular dysfunction, presence of mitral regurgitation and tricuspid regurgitation were analyzed.

### Methodological quality

The methodological quality of the studies was evaluated using the Newcastle-Ottawa scale, a tool indicated for the analysis of cohort and case-control studies. The methodological quality score of the cohort studies was calculated in three components: group selection (0 - 4 points), quality of adjustment for confounding (0 - 2 points) and outcome assessment (0 - 3 points). In case-control studies, group selection (0 – 4 points), quality of adjustment for confounding (0 – 2 points) and exposure (0 – 4 points) were evaluated. The maximum score is 9 points, representing high methodological quality. Two independent researchers judged the quality/risk of bias of the papers.

## Results

### Identification and selection of studies

From the electronic database and manual search, 325 articles were identified. After removing duplicate articles and selecting by reading titles, abstracts and full texts, 4 articles were included in the qualitative synthesis of the work. The selection of studies is represented in the flowchart in Figure 1 .

**Figure 1:** Study selection flowchart.

### General characteristics of studies

Of the four articles selected, three are cohort studies and one is a case-control study. The years of publication ranged from 2016 to 2019. The sample size ranged from 110 to 180 participants, totaling 582 participants: 289 patients with sickle cell anemia, 133 with SC hemoglobinopathy, 40 with beta-thalassemia, 100 healthy individuals and none with alpha-thalassemia or sickle cell trait. Twenty individuals had other sickle cell disease genotypes that did not meet the inclusion criteria for this study. Table 1 presents the general characteristics of the studies.

**Table 1:** General characteristics of the selected studies

Author	Year of publication	Title	Performance period	Place of performance	Study design	Sample size	Objective
Philippe M. Adjagba, Gaston Habib, Nancy Robitaille, et al	2016	Impact of sickle cell anaemia on cardiac chamber size in the paediatric population	Not mentioned	Canada	Retrospective cohort	n = 110	To describe the extent of myocardial abnormalities and determine hematologic indices that could primarily affect cardiac function in patients with sickle cell disease
Jamie K. Harrington, Usha Krishnan, Zhezhen Jin, et al	2017	Longitudinal Analysis of Echocardiographic Abnormalities in Children With Sickle Cell Disease	1994 - 2013	United States	Retrospective cohort	n = 172	To identify clinical and laboratory parameters associated with the development of cardiac abnormalities
Paul Guedeney, François Lionnet, Alexandre Ceccaldi, et al	2018	Cardiac manifestations in sickle cell disease varies with patient genotype	Maio 2008 Maio 2015	France	Retrospective cohort	n = 180	To describe cardiac remodeling and its correlations in patients with HbSC and to compare with patients with sickle cell anemia and with healthy individuals
Antoine Fakhry AbdelMassih, Khaled M. Salama, Carolyne Ghobrial, et al	2019	Discrepancy in patterns of myocardial involvement in beta-thalassaemia vs. sickle cell anaemia	April 2017 October 2018	Egypt	Case control	n = 120	To compare left ventricular mechanics in thalassemia and sickle cell patients using the latest 2D spot tracking analysis software

## Results

Adjagba et al. found that, although right ventricular dilatation was similar between HbSS and HbSC patients, left ventricular dilatation was more frequent in sickle cell anemia than in SC hemoglobinopathy, having been observed in 51.4% vs 24.2% of patients, respectively [OR=2.1(1.11−4.03)] , the same occurring with dilatation of both ventricles, present in 38.9% x 12.5% of patients with each genotype, respectively [OR=3.4(1.19−8.13)] . No significant differences were observed between genotypes in the frequency of left myocardial dysfunction measured by left ventricular shortening fraction and E/e´ ratio. Left ventricular hypertrophy (LVH) was observed in 25% of patients with SCA, which was not observed in the sample with HbSC.

Harrington et al. evaluated 829 echocardiograms performed in 172 patients, and observed a cumulative incidence of echocardiographic abnormalities. The mean age at the first electrocardiogram was 8.74 ± 3.49 years of age (ranging from 5.12 to 19.7 years of age), with a mean of 4.82 ± 3.06 studies performed per patient over a period of 6.88 ± 5.16 years. The age distribution of the first echocardiogram was: 78 (45.4%) aged 5 to 7 years or younger, 72 (41.8%) aged 7 to 13 years or younger, and 22 (12.8%) above 13 years of age. LVH, increased left ventricular end-systolic diameter (LVESD) and left ventricular end-diastolic diameter (LVEDD) were found at an earlier age than abnormal tricuspid regurgitation velocity (TRV), this last one found mainly in late childhood and at the beginning of the adolescence. The prevalence of echocardiographic abnormalities was 25%, 41%, 58%, 7%, and 25% for LVH, increased LVSD and LVDD, decreased LV EF, and increased TRV, respectively. In addition, patients with HbSS and HbSβ0-thalassemia were 8.04% more likely to have LVH, 8.37% more likely to have LV dilatation at the end of systole, and 11.9% more likely to have LV dilatation at the end of diastole. The chance of developing increased tricuspid regurgitation velocity and decreased LV fractional shortening were similar between the genotypes involved in the study.

Guedeney et al. compared cardiac remodeling between individuals with HbSS and HbSC hemoglobinopathies and healthy individuals, involving 180 patients. LV dilatation was greater in patients with HbSS than in subjects with [ $\mathrm{LVDD}/\mathrm{BS}\;=\;32\;\mathrm{mm}/m^{2}\;(\mathrm{IQR}:\;29-33)\;x\;28\;\mathrm{mm}/\;m^{2}\;(\mathrm{IQR}:\;26-30)$ , respectively, p < 0 .0001; $\mathrm{LVEDV}/\mathrm{BS}\;=\;91\;\mathrm{mL}/m^{2}\;(\mathrm{IQR}:\;73-105)\;x\;64\;\mathrm{mL}/m^{2}\;(\mathrm{IQR}:\;54-72)$ , respectively, p < 0.001], the same occurring with AE [ $LAV/SC=49mL/m^{2}(IIQ:42-60)\times 33ml/m^{2}(IQ:30-38)$ , respectively, p < 0.001]. Likewise, LVH was more frequent in SCA than in HbSC [ $\mathrm{MVE}/\mathrm{SC}\;=\;101\;g/m^{2}\;(\mathrm{IQR}:\;84-115)\;x\;76\;g/m^{2}\;(\mathrm{IQR}:\;65-87)$ , p < 0.001; LVM/H = 39 g/m (IQR: 24-48) x32g/m(IQR: 28-36) , p < 0.001] , regardless of the indexing method (body surface or height), noting that the LVH was mostly eccentric. In patients with HbSS, an increase in pulmonary systolic blood pressure – assessed by TRV – was observed in 32 (53%) patients, similar between HbSC patients and controls. LV diastolic dysfunction was more prevalent in SCA than in HbSC and healthy subjects (p = 0.04). Left ventricular ejection fraction (LVEF) was similar in the three groups.

AbdelMassih et al. evaluated the pattern of myocardial involvement in 120 patients in a case-control study. Myocardial T2* was more indicative of myocardial iron overload in patients with beta-thalassemia major than in those with SCA (myocardial T2* = 16.6 ± 1.8 ms; 25.5 ± 2.2 ms, respectively). The global longitudinal strain (GLS) was similar between patients with beta-thalassemia major and those with SCA, but both groups had lower GLS values when compared to healthy individuals (GLS = -15 ± 1.6%; -21.5 ± 1.9%, individuals with beta-thalassemia major and healthy, respectively; GLS = -15 ± 1.2%; -21.5 ± 1.9%, individuals with SCA and healthy, respectively). There was a difference between the groups of hemoglobinopathies hen the epicardial and endocardial GLS were evaluated: the epicardial GLS was lower in patients with beta-thalassemia major (epicardial GLS = -10.9 ± 2%; -19.9 ± 1.7%, in the beta-thalassemia major and SCA, respectively), endocardial SGL was lower in sickle cell patients (endocardial GLS = -19.95 ± 1.7%; -10.65 ± 1.6%, in beta-thalassemia major and SCA, respectively ). It was found that systolic function by LVEF assessed by M-mode and LV fractional shortening was normal and similar in the 3 groups of patients (LVEF = 73.2 ± 3.3 %; 71.2 ± 1.7; 72 .4 ± 2.9, in the beta-thalassemia major group, SCA and healthy individuals, respectively; LV shortening fraction = 35.5 ± 2%; 35.5 ± 0.98%; 37.5 ± 3.3% , in the beta-thalassemia major group, SCA and healthy individuals, respectively), the same being observed for LV diastolic function by the E/e´ ratio (E/e´ = 6.89 ± 2; 6.6 ± 1.9 ; 6.52 ± 1.49 in the beta-thalassemia major group, SCA and healthy subjects, respectively). LVEF assessed by the 3D mode was lower in patients with SCA than in controls ( LVEF=62%±11.2×66%±13.2 , respectively) and also lower in patients with beta-thalassemia greater than in controls LVEF=61%±10.1×66%±13.2 , respectively), being similar in both hemoglobinopathies. The main results are shown in Table 2 .

**Table 2:** Main results of the selected studies

Author/year of publication	Population	Analyzed cardiovascular parameter	Cardiovascular alteration found	Conclusion
Philippe M. Adjagba et al. (2016)	110 patients with sickle cell disease (72 HbSS; 32 HbSC; 6 HbSβ – thalassemia)	RVV LVV LVM FSLV E/e´ relation MPI	RVD LVD Diastolic dysfunction Systolic dysfunction Abnormality in LVM	LVD was higher in patients with SCA (HbSS) than in patients with SC hemoglobinopathy (HbSC); LVH was observed only in patients with SCA (HbSS) and the abnormality in LVM was more prevalent in this group of patients; RVD, FSLV and E/e´ relation were similar between the patients with SCA (HbSS) and the individuals with SC hemoglobinopathy (HbSC).
Jamie K. Harrington et al. (2017)	172 patients with sickle cell disease (117 HbSS; 41 HbSC; 5 HbSβᴼ – thalassemia; 9 HbSβ⁺ – thalassemia)	LVM LVESD LVEDD FSLV TRV	LVH LVD at the end of the systole and at the end of the ao final da sístole e ao final da diastole ↓EF OF LV ↑TRV	Patients HbSS e HbSβ0-thalassemia were more likely to develop LVH, LVD at the end of the sistole and at the end of the diastole. The chance of developing increased TRV and decrease of FSLV were similar between all genotypes involved in this study.
Paul Guedeney et al. (2018)	120 patients with sickle cell disease (60 HbSS; 60 HbSC) and 60 healthy patients	LVEDD/BS LVM/BS LVM/H LVEDV/BS CI TRV EFLV EM wave A wave E/A relation DT e´ wave E/e´ relation LAV/BS	LVD at the end of diastole LAD LVH ↑CI ↑TRV ↑E/e´relation LVDD	LAD, LVD e CI were higer in HbSS patients than in HbSC patients and the controls; LVH, increase of TRV and diastolic dysfunction disfunção of LV were more frequent in HbSS patients than in HbSC patients and the controls (HbSS patients had higher: E wave, E/A relation, DT, e´wave, E/e´ relation); LAD, LVD, LVM/BS, LVM/H, E/e´ relation were higer in HbSC patients than in the control; e´ wave was smaller in HbSC patients than in controls; CI e TRV were similar between HbSC patients and the controls; EFLV was similar between the 3 groups.
Antoine Fakhry AbdelMassih et al. (2019)	40 patients with sickle cell anemia (HbSS), 40 patients with beta thalassemia major (β0/β0) and 40 healthy patients	EFLV FSLV E/e´ relation GLS GLS epicardial SGL endocardial T2* miocardyal	Myocardial iron overload ↓GLS Subendocardial dysfunction Subepicardial dysfunction	T2* myocardial was higher in patients with beta thalassemia major than in patients with SCA; GLS was similar between patients with beta thalassemia major and those with SCA, but both groups of patients had GLS reduced in comparision with healthy individuals; Epicardial GLS was lower in patients with beta thalassemia major than in patients with SCA; Endocardial GLS was lower in patients with SCA than in patients with beta thalassemia major; The systolic function and the diastolic function of left ventricle were normal and similar between the 3 groups.

A wave: Wave of atrial contraction in mitral flow; CI: Cardiac index; DT: Deceleration time; e´ wave: Tissue Doppler early diastolic velocity wave; E/e´ relation: Relation between E wave in the mitral flow and the e wave by tissue Doppler; E/A relation: Relationship between E and A waves in mitral flow; EF: Ejection fraction; EFLV: Ejection fraction of left ventricle; EM wave: Rapid filling wave in mitral annulus mitral flow; FSLV: Fraction of shortening of the left ventricle; GLS: Global longitudinal strain; LAD: Left atrial dilatation; LAV/BS: Left atrial volume indexed by body surface; LV: Left ventricle; LVD: Left ventricular dilatation; LVDD: Left ventricular diastolic dysfunction; LVEDD: Left ventricular end-diastolic diameter; LVEDV/BS: Left ventricular end-diastolic volume indexed by body surface; LVEF: Left ventricular ejection fraction ; LVH: Left ventricular hypertrophy; LVESD: Left ventricular end-systolic diameter; LVM: Left ventricular mass; LVM/BS: Left ventricular mass indexed by body surface; LVM/H: Left ventricular mass indexed by height; LVV: Left ventricular volume; MPI: myocardial performance index; RVD: Right ventricular dilatation; RVV: Right ventricular volume; TRV: Tricuspid regurgitation velocity; T2*: Myocardial relaxometry of cardiac magnetic resonance.

### Risk of bias of selected studies

The methodological quality of the studies included in this review was high. Of the cohort studies, one scored eight points on the Newcastle-Ottawa scale and two scored nine points on the same scale. The case-control study obtained 8 points on the scale used.

## Discussion

Cardiovascular complications are the main cause of morbidity and mortality in patients with HbSS. The role of the echocardiogram for the early identification of cardiac alterations in these patients is highlighted, as evidenced by the findings of this study. Thus, a higher prevalence of ventricular hypertrophy, dilatation of cardiac chambers, diastolic dysfunction, mitral and tricuspid regurgitation and pulmonary hypertension was observed in individuals with sickle cell anemia compared to those with the other hemoglobinopathies considered in this study.

Dilatation of the cardiac chambers, especially the LV, results from compensatory myocardial remodeling in response to chronic anemia.^7 - 12^ The analysis of associations between echocardiographic variables in patients with sickle cell disease showed that individuals with higher LVDD/BS had higher LAV/BS and TRV values, as well as lower LVEF, indicating left systolic dysfunction with repercussions in the right chambers.^2 , 10^ LVH was independently associated with changes in the echocardiographic parameters of diastolic dysfunction, such as a decrease in the deceleration time of the early mitral inflow velocity, an increase in the E/e´ ratio and an increase in the velocity of tricuspid regurgitation, which can be explained by the reduction in left ventricular compliance in these patients.

Diastolic dysfunction is among the main cardiovascular alterations reported in sickle cell disease, and the frequency of this finding depends on the echocardiographic parameters used to assess diastolic function, the patient’s age and associated comorbidities.^9^ Vasconcelos et al.^9^ explained the occurrence of normal diastolic function in individuals with sickle cell disease as a result of a young age (mean age of 26.5 years), absence of comorbidities and use of tissue Doppler, whose greater specificity derives from its ability to measure myocardial velocities, not suffering alterations with preload changes.^13^

The association verified by Whipple et al.^14^ between decreased e´M and e´T and also decreased LVGLS and RVGLS, suggests that the increased prevalence of diastolic dysfunction in children with sickle cell disease reduces myocardial deformability, measured by the GLS. In patients with HbSC, while Adjagba et al.^7^ observed a similar E/e´ ratio between these patients and individuals with HBSS, Guedeney et al.^15^ found a higher frequency of left ventricular diastolic dysfunction in patients with SCA and systemic arterial hypertension, which corroborates the hypothesis suggested by Desai et al.^8^ that the impairment of diastolic function in this group of patients results from increased afterload. These data suggest that diastolic dysfunction is frequent, early and likely to have a multifactorial etiology in individuals with SCA.

In patients with sickle cell disease, systolic function is normally preserved. However, a significant prevalence of low left ventricular systolic function has already been demonstrated in patients with HbSS and HbSC.^7^ An early marker of systolic dysfunction, the GSL measures myocardial deformability, and the increase in its values indicates the existence of a baseline condition altering myocardial deformability as a compensatory mechanism. When evaluating the association of GLS with traditional measures of ventricular systolic function – LVEF and STTAP – in children with sickle cell disease, Whipple et al.^14^ showed agreement between these variables: decreased LVGLS and RVGLS associated with decreased LVEF and STTAP. The decrease in STTAP reflects impaired RV systolic function. As LV systolic function is usually preserved in sickle cell disease, abnormal STTAP may indicate chronic elevation of pulmonary pressures. Furthermore, the RVGLS was impaired by high pulmonary pressure and RV diastolic dysfunction.^12^

In the comparison between SCA and beta-thalassemia major, the included case-control study^16^ showed a predominance of subendocardial dysfunction in SCA and subepicardial dysfunction in beta-thalassemia major, explained by the high vascularity of the epicardium with consequent iron deposition. Myocardial T2* was strongly correlated with epicardial GLS but not with endocardial GLS. In turn, the decrease in subendocardial GLS seen in SCA is justified by the microvascular disease in these patients, characterized by possible subendocardial microvascular ischemia, through NO depletion and suggested by the increase in LDH.

Regarding the parameters to assess systolic function, it is worth mentioning that, in the same study, LVEF measurements differed according to the method used: when evaluated by the M-mode, LVEF was similar between the 3 groups, however, when analyzed by 3D echocardiography, LVEF was shown to be lower in individuals with SCA than in healthy individuals and similar in comparison with those with major beta-thalassemia.

Right ventricular function is commonly assessed through TRV and systolic excursion of the tricuspid annulus plane. TRV was included among the predictors of adverse events in the work by Vasconcelos et al.^9^ Furthermore, TRV ≥ 2.5 m/s was a predictor of mortality within 3 years by Damy et al.^10^ In this work, elevated TRV was associated with lower LVEF and higher LAV/BS, changes commonly associated with high filling pressures and the risk of postcapillary pulmonary hypertension.

It is noteworthy that most studies were carried out with relatively small samples. In addition, the cardiovascular variables analyzed differed in the included studies. Despite the limitations, the present review should be considered an update tool on a pathology of systemic involvement, allowing a better understanding of cardiovascular alterations in the different genotypes of hemoglobinopathies.

## Conclusion

The prevalence of cardiovascular complications such as cardiac chamber dilatation, LVH and RVH, pulmonary hypertension, diastolic dysfunction, mitral regurgitation and tricuspid regurgitation are higher in patients with SCA than in individuals with the other hemoglobinopathies considered in this study. Overall, there were no differences between the systolic function of patients with SCA and those with other hemoglobinopathies.
